# The impact of brief educational intervention on Jordanian community pharmacists’ knowledge, attitudes, and practices in asthma management: A quasi-experimental study

**DOI:** 10.1371/journal.pone.0356821

**Published:** 2026-08-27

**Authors:** Mahmoud S. Abdallah, Rania Mahafdeh, Rima Hisham Shakhatreh

**Affiliations:** 1 Department of PharmD, Faculty of Pharmacy, Jadara University, Irbid, Jordan; 2 Department of Pharmacy, Faculty of Pharmacy, Jadara University, Irbid, Jordan; Isra University Faculty of Pharmacy, JORDAN

## Abstract

**Background:**

Community pharmacists play a pivotal role in asthma self‑management support. Nonetheless, important care gaps persist.

**Aim:**

This study aimed to evaluate the impact of an educational intervention on knowledge, attitudes, and practices (KAP) scores among community pharmacists regarding asthma management.

**Methods:**

A quasi‑experimental two‑week educational intervention study was conducted among 110 licensed Jordanian community pharmacists. Baseline knowledge, attitudes, and practices (KAP) regarding asthma management were collected using a validated questionnaire. Participants then attended a guideline-aligned 2-hour educational session. The same questionnaire was administered for two weeks post‑intervention. Univariate and multivariate analyses were employed to determine the associated factors for baseline KAP scores. Changes in KAP scores were analyzed using the Wilcoxon signed‑rank test, and changes in proportions of correct knowledge responses were assessed with McNemar’s test.

**Results:**

A total of 110 community pharmacists took part in the study, and 100 completed the post-intervention assessment. Education level was significantly associated with pharmacists’ KAP. Pharmacists holding PharmD degrees had higher KAP scores compared with those holding a BSc in Pharmacy (p < 0.05). After the educational program, knowledge, attitude, and inhaler-use practice scores (MDI and DPI) were associated with short-term improvements in self-reported practice from before to after the intervention in all analyses (p < 0.001), showing improvement in pharmacists’ knowledge, attitude and practices scores after the training.

**Conclusion:**

Following the short guideline‑based workshop, community pharmacists showed improvements in knowledge scores, attitudes toward asthma care, and self‑reported inhaler‑use practices. These findings suggest that a brief educational intervention may be associated with short‑term enhancements in pharmacists’ asthma‑related competencies.

## 1. Introduction

Asthma is a significant worldwide health issue affecting people of all ages. The prevalence of asthma in Jordan is high, ranging from 8.8% to 9.5% among children in one study, which also showed that the prevalence of asthma has alarmingly doubled over ten years in Jordan [1]. Further recent studies revealed a prevalence of asthma of 2.38% among schoolchildren [2] and reaching 10% among elderly patients in Jordan [3].

The overall yearly cost of asthma in Jordan was estimated to be 110,874 Jordanian dinar (JD) (US$ 156,382), with healthcare resources utilization and direct medical expenditures of asthma being substantially associated with disease severity and disease control status [4].

In most patients, control can be achieved through appropriate therapy, including: inhaled corticosteroids (ICS), combination ICS/long-acting beta2-agonists (LABA), “triple therapy” with ICS/LABA/long-acting muscarinic receptor antagonist (LAMA), and biologic therapies [5]

Many factors influence the success of treatment and the achievement of disease control, such as asthma triggers, comorbidities and asthma phenotype [6]. Other influences may be related to the physician (e.g., not following guidelines, assessing the patient inappropriately, or inadequate selection of treatment or inhaler device) or the patient (e.g., sociodemographic factors, exposure to cigarette smoke, poor treatment adherence, and inadequate inhaler technique or disease education) [7,8]. Additional contributing factors may relate to the healthcare system, such as medication coverage and spirometry testing [9].

Pharmacists can play a significant role in the management of chronic diseases including asthma [10]. Due to their clinical expertise, pharmacists are well-equipped to educate patients with asthma about their medical condition, explain the mechanisms of asthma medications, demonstrate correct inhalation techniques, address any concerns regarding potential side effects, and promote adherence to prescribed therapies [11]. This positions them uniquely to contribute to optimal clinical outcomes for individuals with asthma [11].

Pharmacists’ interventions in asthma care are highly encouraged in practice; however, the level of these interventions to be considered minimal or standard is not yet clearly specified [12]. Moreover, the wide variability in pharmacists’ knowledge, attitudes, and practices in asthma may not be fully consistent with GINA recommendations [13].

Management of asthma requires teamwork from patients, health-care providers, and pharmacists who are among the most accessible health professionals for patients [11]. Community pharmacists can greatly contribute to the management and control of asthma by providing sufficient education to patients about their medications [14]. Improving community pharmacists’ knowledge, attitudes, and practices through evidence-based practical, local educational interventions may positively impact patient care [15].

Studies indicate specific areas in community pharmacists’ knowledge, attitude, and asthma counseling practice that need improvement [16–18]. To tackle these areas effectively, it is crucial to involve pharmacists in educational and training programs that focus on asthma care [19]. Moreover, the development and implementation of continuing education programs that equip pharmacists with updated asthma management guidelines, treatment options, and counseling techniques are essential [20].

The impact of educational interventions on community pharmacists’ knowledge, attitudes, and practices; as well as factors associated with their effectiveness, has been extensively documented in many countries; however, in Jordan, studies remain scarce [21]. Several programs have been delivered to pharmacists after graduation, reflecting a growing interest in the role of continuing professional development (CPD), including education-focused programs aimed at addressing the determinants of professional behavior change and promoting practice adaptation [16]. Community pharmacists play an important role in asthma care, and their knowledge, attitudes, and daily practices are critical, highlighting the need for more education and training [18]. However, a considerable gap has been reported in Jordanian pharmacists’ knowledge, attitudes, and practices concerning asthma management [18].

Therefore, this study aimed to assess the current level of knowledge, attitudes, and practices of Jordanian community pharmacists regarding asthma management and the factors associated with them. Furthermore, it aimed to evaluate the impact of an educational intervention on knowledge, attitudes and practices among community pharmacists regarding asthma management.

## 2. Methods

### 2.1 Study design

This study employed a quasi‑experimental pre–post design. The study was conducted among community pharmacists in Jordan. Data were collected from June 1, 2025, to November 30, 2025.

### 2.2 Ethical considerations

Ethical approval for this study was obtained from the Institutional Review Board (IRB) of the Faculty of Pharmacy, Jadara University (PHARM-JA-23/2025). Written informed consent was obtained from all participants before their enrollment, ensuring voluntary participation. The confidentiality and anonymity of participants’ responses were maintained throughout the study. No identifiable personal information was collected or reported.

Limited contact information was collected solely for communication and follow-up purposes and was not linked to participants’ responses. All data were handled securely and used exclusively for research purposes.

### 2.3 Inclusion and exclusion criteria

The inclusion criteria included licensed pharmacists currently practicing in Jordan, engaged in dispensing or counseling on respiratory medications, and willing to participate in both pre- and post-intervention assessments. Pharmacists with prior advanced respiratory pharmacotherapy training and those unable to complete follow-up assessments were excluded from the study.

### 2.4 Sample size calculation

Using a two-sided McNemar test with a significance threshold of 0.050, a sample size of 92 provided 80.138% power to detect an odds ratio of 4, assuming approximately 20% improvement among participants [22,23]. The odds ratio represents the difference between the two paired proportions: 0.150 and 0.250. Assuming a 20% dropout rate, the overall sample size was 110.

### 2.5 Data collection tool and outcomes

A validated knowledge, attitudes, and practices (KAP) questionnaire was administered before and after an educational intervention [16]. The questionnaire, which was based on a previously validated instrument, underwent final minor adaptations prior to data collection. These included the modification of one knowledge item and adjustments in the practice section to match MDIs and DPIs, while the attitude scale remained unchanged. It included: Knowledge Section (7 items): assessing knowledge about asthma management. The knowledge score ranged from 0–7. Attitude Section (5 items): assessing attitudes towards asthma management and counselling. Participants rated their level of agreement on a 5-point Likert scale (from Strongly Disagree to Strongly Agree). The attitude score ranged from 0–25. Practice Section (22 items per device): The practice domain assessed asthma counseling behaviors separately for Metered-Dose Inhalers (MDIs) and Dry-Powder Inhalers (DPIs). Each device-specific section included both technique-related items and general asthma counseling behaviors (e.g., adherence assessment, action plan review, and follow-up). These general items were intentionally repeated across both sections to evaluate the consistency of counseling practices within each device context. MDI and DPI practice scores were calculated independently, and no items were double counted within a single domain score. Participants reported how often they provided each service using a 5-point Likert scale (ranging from Never to Always). The practice score ranged from 0–110.

Content validity was assessed through an expert panel review involving five subject matter experts, including clinical pharmacists and academic staff with experience in asthma management, questionnaire development, and pharmacy practice research. The panel evaluated each item for relevance, clarity, and clinical appropriateness.

A pilot study (n = 20) was then conducted using the finalized version of the questionnaire to assess clarity and internal consistency. As no further modifications were required following pilot testing, pilot participants were included in the final analysis. All participants completed the questionnaire only once, with no re-administration or re-exposure to different versions of the instrument. To minimize potential recall bias, pilot participants were not provided with feedback on their responses, and the main study data collection was conducted after a sufficient interval.

Internal consistency was evaluated in both the pilot and the full sample. In the pilot phase, the reliability values were 0.881 for knowledge (Cronbach’s alpha, equivalent to the Kuder–Richardson Formula 20 (KR-20) for dichotomous items), 0.720 for attitude, 0.926 for MDI practice, and 0.922 for DPI practice. In the full sample (n = 110), reliability was recalculated using all responses and showed values of 0.696 (standardized α = 0.711) for knowledge, 0.611 (standardized α = 0.624) for attitude, 0.961 (standardized α = 0.963) for MDI practice, and 0.956 (standardized α = 0.960) for DPI practice.

### 2.6 Recruitment strategy

Community pharmacists working in different regions of Jordan were recruited through advertisements posted on professional social media platforms, particularly Facebook groups targeting licensed pharmacists in Jordan. Pharmacists who were self-selected to participate in the study by responding to the social media advertisement were directed to a closed group created for the purpose of this study on a social media platform (WhatsApp) to further arrange for their participation. Eligible participants provided written informed consent prior to enrollment and were then added to a closed WhatsApp group created specifically for study coordination, through which workshop logistics and follow‑up reminders were communicated.

Participants were invited to attend a two‑hour educational workshop, and participation in both the workshop and the study assessments was entirely voluntary. Data were collected using a structured, self‑administered questionnaire consisting of two main sections: demographic and socioeconomic characteristics, knowledge, attitudes, and practices (KAP) related to asthma management. The questionnaire was designed as a self-administered tool and participation was entirely voluntary. The KAP questionnaire was administered again two weeks after the educational session.

### 2.7 Educational workshop

The educational workshop was delivered through 11 face-to-face sessions; each was held with a different group of 10 participants. Each session lasted around two hours and followed a standardized format to maintain consistency across sessions. PowerPoint presentations, videos, and clinical case scenarios were used.

A structured health education session was prepared and revised by a specialized clinical pharmacist according to GINA (2025). A structured PowerPoint presentation in English with a printout was used to deliver the health education program. The presentation’s contents were organized in the following order: a common definition of asthma, causes, risk factors, diagnosis, treatment modalities and algorithms, use of inhalers, preventive measures, and a final message.

All sessions used the same material, structure, and delivery approach. The participants were asked not to provide the materials outside the sessions and were asked not to share the workshop content or questionnaire details with others until the study was completed.

### 2.8 Data analysis

Data were analyzed using descriptive and inferential statistical methods. Descriptive statistics, including frequencies, percentages, medians, and interquartile range (IQR) were used to summarize the characteristics of the participants and their responses. Normality of KAP scores was checked using the Kolmogorov–Smirnov test. The results showed that the scores were not normally distributed. Therefore, the Mann-Whitney U test was used to examine the relationship between continuous variables, such as KAP score, and sociodemographic factors. A correlation test using the Spearman correlation coefficient was utilized for testing the relationship with age, working experience (years), weekly working hours, number of patients served per day, number of prescriptions dispensed per day, number of pharmacists working together, and time spent with each patient (in minutes).

Variables included in the multivariate model were selected based on findings from univariate analysis and theoretical relevance. Variables with a p-value < 0.20 in univariate analyses and those considered clinically or theoretically relevant were entered into the multivariable models. Age and years of experience were highly correlated; therefore, only years of experience were included in the regression analysis.

Poisson regression was employed to identify factors associated with knowledge scores, which is appropriate for the count nature of the dependent variable. Beta coefficients (β) and rate ratios (RR) were reported to describe associations. The Wald χ² test assessed the significance of predictors. 95% Confidence Intervals (CI) were provided for all RRs. The Poisson regression model fitted the data well, with no evidence of overdispersion (Pearson χ²/df = 0.563 and deviance/df = 0.685).

Linear regression analysis was employed to identify independent predictors of attitudes and practices outcomes. Variables included in the multivariate model were selected based on findings from univariate analysis and theoretical relevance. Attitude and practice outcomes were significantly explained by the model. Attitude scores were explained by 19.2% of the variance (R² = 0.192, p = 0.009). The MDI practice score explained 33.8% of the variance (R² = 0.338, p < 0.001), while the DPI practice score explained 33% (R² = 0.33, p < 0.001). Residuals showed no autocorrelation (Durbin–Watson ranged from 1.879 to 2.037), and multicollinearity was not a concern (VIF ≤ 1.328).

The Wilcoxon signed-rank test was used to compare pre- and post-intervention KAP scores. The changes in the proportion of correct responses in knowledge were compared using McNemar’s test. Multiple comparisons for item-level McNemar tests were adjusted using the Benjamini–Hochberg procedure to control the false discovery rate (BH-FDR). Attitude and practice were presented using descriptive statistics and compared as total scores only, as some response categories disappeared after intervention, making McNemar or Pearson’s chi-square test unreliable.

To assess whether missingness may be related to baseline characteristics, we compared completers and non-completers using Mann–Whitney U tests for continuous variables and Fisher’s exact test for categorical variables.

Analyses were conducted using both the per-protocol (PP) and intention-to-treat (ITT) approaches. Missing data were handled using multiple imputations with 10 imputed datasets for the intention-to-treat analysis. Baseline characteristics of completers and non-completers were compared using Mann–Whitney U tests for continuous variables and Fisher’s exact test for categorical variables. Effect sizes were recalculated using rank-biserial correlation (r(rb)) for all Wilcoxon signed-rank tests to provide a standardized and interpretable measure of the magnitude of pre–post changes. A p-value of <0.05 was considered statistically significant. All analyses were conducted using SPSS (version 27).

## 3. Results

### 3.1 Study participants

**Fig 1:**
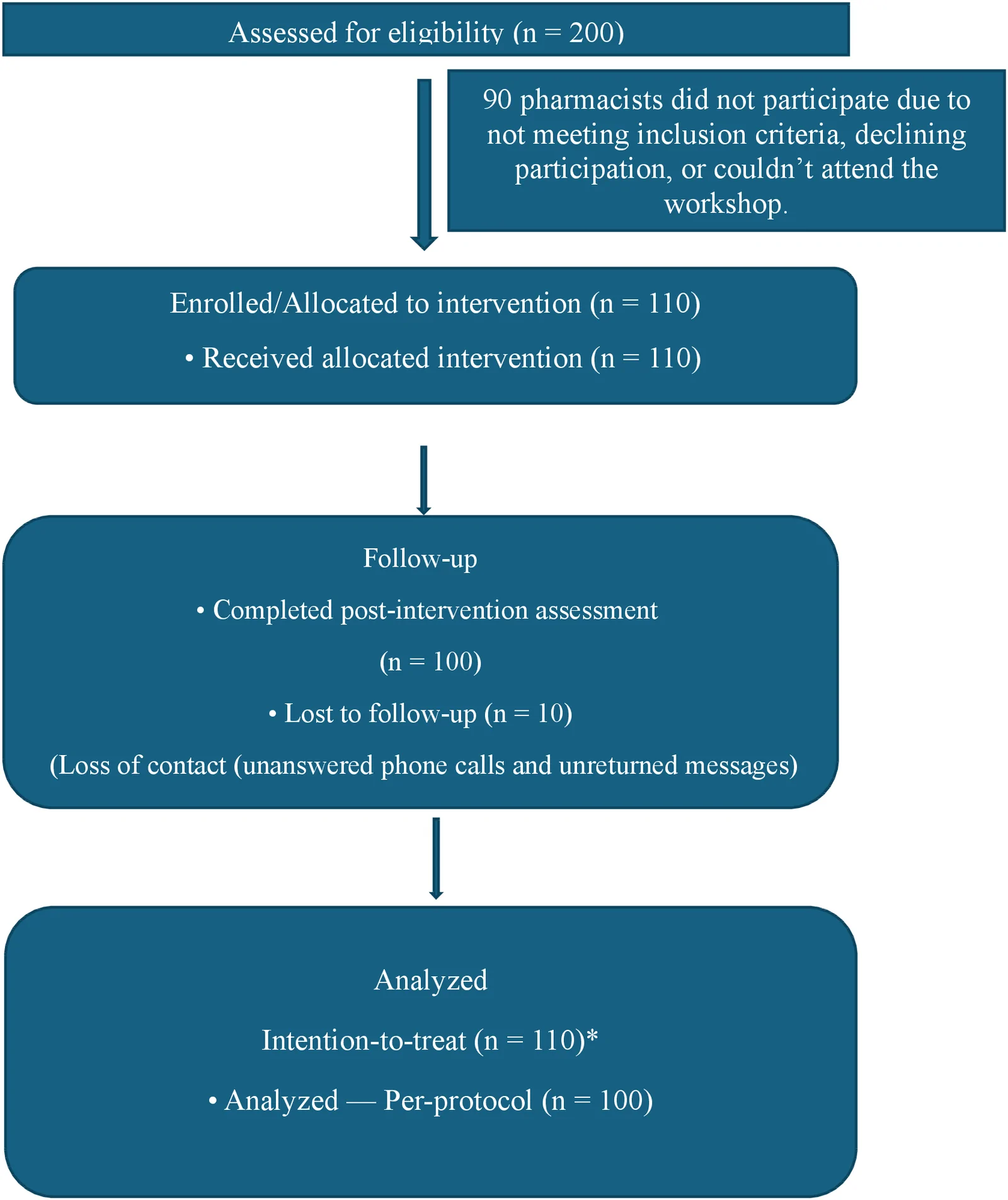
Flow chart of included study participants in the study.

A total of 200 pharmacists responded to the recruitment announcement and were assessed for eligibility. Of these, 110 met the inclusion criteria and consented to participate in the study, while the remaining pharmacists were either ineligible (e.g., prior advanced respiratory pharmacotherapy training) or declined participation (Fig 1).

All enrolled participants completed the pre‑intervention assessment and attended the educational workshop. During the two‑week follow‑up, 100 participants completed the post‑intervention assessment, while 10 participants were lost to follow‑up due to loss of contact despite repeated reminders (including unanswered phone calls and unreturned messages).

A comparison of baseline demographic characteristics and pre‑intervention KAP scores between participants who completed follow‑up and those lost to follow‑up did not reveal any meaningful differences, suggesting limited attrition‑related bias.

### 3.2 Sociodemographic and basic data of the study subjects

As shown in Table 1, the median age of the pharmacists was 27 years (IQR 25–31), and most had limited work experience, with a median of 4 years (IQR 3–8). They worked long hours, with a median of 54 hours per week, and each pharmacist served a median of 30 patients per day, dispensing about 10 prescriptions daily (IQR 10–15). Most pharmacies had only 1 or 2 pharmacists working together, usually the participant and one colleague. The median time spent with each patient was 5 minutes. Regarding demographics, 38.2% were male and 61.8% were female. About 52.7% had a Bachelor of Science in Pharmacy and 47.3% had a PharmD, while around 32.7% held postgraduate degrees. More participants were from urban Amman than Irbid, and pharmacies were either independent (51.8%) or part of a chain (48.2%). All participants had access to the internet.

**Table 1:** Sociodemographic and basic data of the study subjects (n = 110).

Variable	Median	Interquartile range (IQR)
**Age**	27	(25-31)
**Working experience (years)**	4	(3-8)
**Weekly working hours**	54	(45-54)
**Number of patients served a day**	30	(20-40)
**Number of prescriptions dispensed per day**	10	(10-15)
**Number of pharmacists working with**	1	(1–2)
**Time spent with each patient (in minutes)**	5	(5-8.25)
	**Frequency (n = 110)**	**%**
**Gender**		
**Males**	42	38.2
**Females**	68	61.8
**Education background**		
**Pharmacy**	58	52.7
**Pharm D**	52	47.3
**Post graduate degree**		
**Yes**	36	32.7
**No**	74	67.3
**Location of pharmacy**		
**Irbid**	34	30.9
**Amman**	76	69.1
**Pharmacy type**		
**Chain community pharmacy**	53	48.2
**Independent community pharmacy**	57	51.8
**Access to the internet**		
**Yes**	110	100
**No**	0	0

### 3.3 Pre-intervention assessment of participants’ knowledge, attitude, practice regarding asthma management

Before the intervention, participants had varying levels of knowledge about asthma management. Just over half (51.8%) knew that guidelines advise against SABA-only therapy. Knowledge was lowest for safety and pharmacotherapy issues: only 40.9% knew that inhaled corticosteroids do not significantly affect child growth, and 40.0% were aware that using only a SABA increases the risk of severe attacks and asthma-related death. The median total knowledge score before the intervention was 5 (IQR: 4–6) (S1Table).

Most participants had generally positive attitudes toward asthma management before the intervention. However, the confidence in their own ability to manage asthma patients was lower: only 41.8% agreed or strongly agreed, 33.6% were neutral, and 24.5% disagreed. The median pre-intervention attitude score was 19 (IQR: 18–21) (S2Table).

The baseline assessment of MDI counseling practices. Overall, participants showed a moderate level of practice. Most participants reported that they sometimes or often used their inhalers correctly. The median total MDI practice score before the intervention was 77, with an interquartile range of 65–82 (S3 Table). The pre-intervention assessment of DPI counseling practices was similar to MDI counseling, with most participants performing technical inhaler steps sometimes or often. The median pre-intervention total DPI practice score was 75 (IQR: 65–82) (S4 Table).

### 3.4 Univariate analysis for factors affecting the pre-intervention knowledge, attitude, and practice scores

The pre-intervention knowledge scores of pharmacists varied depending on education and experience, with little impact from practice setting. PharmD graduates achieved substantially higher scores than BSc Pharmacy (median 6 vs 4, p < 0.001). There was a moderate positive correlation between pharmacists’ age and their knowledge of asthma management (r = 0.466, p < 0.001), as well as between years of professional experience and knowledge (r = 0.381, p < 0.001). A weaker positive correlation was observed with the number of prescriptions dispensed per day (r = 0.212, p = 0.026) (S5 Table).

The pharmacists’ pre-intervention attitude scores varied based on their education and type of pharmacy. PharmD graduates scored higher than BSc holders, with a median of 20 versus 18 (p < 0.001). Pharmacists working in chain pharmacies had higher scores than those in independent pharmacies, with medians of 20 versus 19 (p < 0.010). There was a weak but significant positive correlation between pharmacists’ age and their attitude toward asthma management (r = 0.212, p = 0.026), as well as between years of professional experience and attitude (r = 0.255, p = 0.007) (S6 Table).

Knowledge and attitude scores showed no significant differences for postgraduate degree, sex, or city. (p > 0.05).

The baseline MDI and DPI counseling practice scores differed mainly by education and location. PharmD graduates scored higher than BSc Pharmacy graduates, with medians of (81 vs 65), p < 0.001) and 79.5 vs. 65, p < 0.001), respectively. Pharmacists working in Amman also had higher MDI and DPI practice scores than those in Irbid, with medians of 78 vs 65, p = 0.045) and 77.5 vs 65, p = 0.029), respectively. MDI and DPI practice scores showed a positive correlation with age (r = 0.322, p < 0.001) and (r = 0.342, p < 0.001), and years of professional experience (r = 0.285, p = 0.003) and (r = 0.301, p = 0.001), respectively. In contrast, MDI and DPI counseling practice scores showed a weak but significant negative correlation with weekly working hours (r = −0.199, p = 0.037), and (r = −0.206, p = 0.031), respectively. No significant correlations were found between practice scores and patient load, number of prescriptions per day, team size, or time spent with each patient (all p > 0.05). MDI and DPI counseling practice scores showed no significant differences for sex, postgraduate education, or pharmacy type (p > 0.05) (S7 and S8 Tables).

### 3.5 Multivariate analysis for predictors affecting pre-intervention knowledge, attitude, practice scores

According to the Poisson regression model for baseline knowledge, education level was the only factor significantly associated with pharmacists’ knowledge. Pharmacists with a BSc in Pharmacy had a 34% lower expected knowledge score compared with those holding a PharmD (β = −0.414; RR = 0.66; p < 0.001) (S9 Table).

The multiple linear regression analysis of pharmacists’ attitude scores found that education was the only significant predictor of attitude scores. Pharmacists with a higher degree, such as a PharmD compared to a BSc in Pharmacy, scored on average 1.326 points higher in attitude (β = 1.326, SE = 0.58, t = 2.286, p = 0.024). All other factors including postgraduate degree, location, type of pharmacy, years of experience, and number of prescriptions per day, were not significant after adjustment for knowledge and attitude scores (S10 Table).

Regarding linear regression analysis for MDI and DPI practice scores, education had the strongest effect, with PharmD scoring about 6.667 points and 6.27 points higher than those with BSc in pharmacy. In addition, working in Amman also increased scores by about 4.618 and 4.826 points for MDI and DPI practice scores, respectively. More years of work experience had a smaller effect, increasing the practice scores by around 0.4 points for each additional year. Other factors, including having a postgraduate degree, pharmacy type, and number of prescriptions per day, did not significantly affect MDI and DPI practice scores (S11 and S12 Tables).

### 3.6 Pre-Post educational intervention participants’ KAP responses regarding asthma management

**Fig 2:**
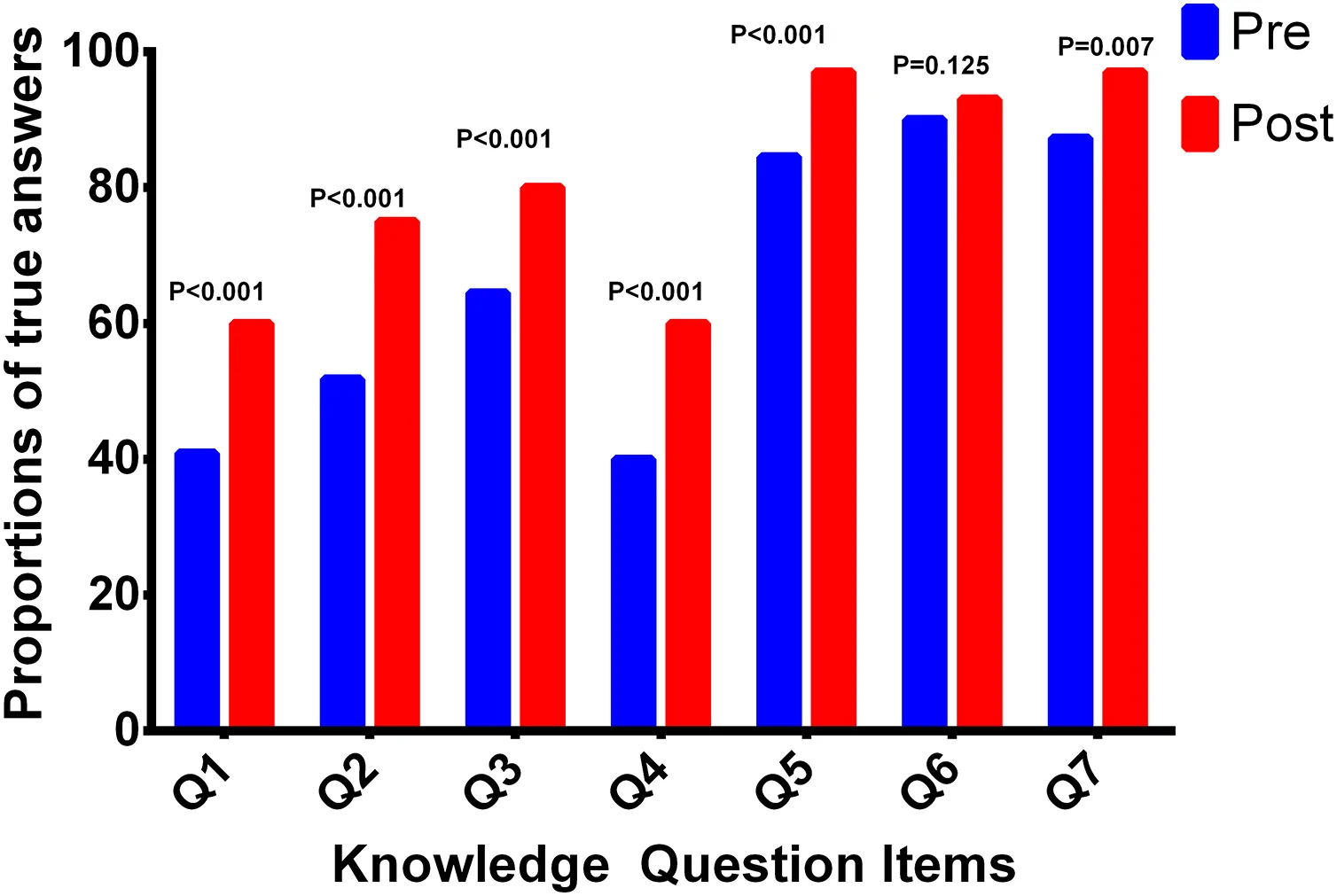
Pre and Post education intervention comparison of knowledge true answer proportions among participants using McNemar’s test corrected using the Benjamini–Hochberg False Discovery Rate (FDR) Procedure.

(Fig 2) shows the McNemar’s test results comparing the proportion of correct answers before and after the workshop for the 7 knowledge items. To control for multiple testing, p-values were adjusted using the Benjamini–Hochberg false discovery rate (BH-FDR) procedure with a significance threshold of *q* = 0.05. Overall, the post-intervention results were higher than the pre-intervention results for all items. Question 1 about the effect of inhaled corticosteroids on children’s growth improved from about 41% to 60%. Question 2 on the fact that current guidelines no longer support using SABA alone increased from about 52% to 75%. Question 3 about inhaled corticosteroid dose levels improved from about 65% to 80%. Question 4 on the risk of severe asthma attacks and death with SABA-only use improved from about 40% to 60%. All these changes were highly significant (p < 0.001). Question 5 about factors causing poor asthma control increased from around 85% to 97%. Question 7 on avoiding nebulizers during epidemics improved from about 87% to 97% (p = 0.007). Question 6 on common asthma triggers showed only a small improvement and was not statistically significant (p = 0.125).

Detailed question statements are provided in S1 Table.

Post-intervention improvements were observed across all attitude items (Q1–Q5). Most participants agreed or strongly agreed that pharmacists are essential members of the asthma care team (Q1), increasing from 49.1% pre-intervention to 95% post-intervention. Confidence in managing asthma patients appropriately (Q2) also improved, with the “agree” responses rising from 38.2% pre-intervention to 51% post-intervention. Regarding the need for continuing medical education (Q3), participants increasingly recognized that pharmacists must participate in additional training to be competent asthma educators, with agree/strongly agree responses increasing from 59.1% and 32.7% pre-intervention to 50% and 50% post-intervention. Similarly, attitudes toward disease monitoring with peak flow meters improved (Q4), with agree responses rising from 46.4% pre-intervention to 75% post-intervention. Finally, participants recognized more strongly that appropriate patient counseling (Q5) has a significant impact on asthma management success, with total agreement (agree and strongly agree) increasing from 85.5% pre-intervention to 100% post-intervention (S1 Fig).

Post-intervention improvements were observed across all MDI counseling practice items (Q1–Q22). For inhaler technique steps, responses shifted toward higher frequencies of “often” and “always” after the workshop. Specifically, Q1 (shaking the inhaler and removing the cap) increased from 48.2% “often” pre-intervention to 60% post-intervention, Q2 (breathing out completely) from 51.8% to 60%, Q3 (holding the inhaler upright) from 51.8% to 60%, Q4 (inserting the mouthpiece correctly) from 51.8% to 60%, Q5–Q6 (coordinating actuation with slow, deep inhalation and completing the inhalation) from 51.5–51.8% to 60%, and Q8 (holding breath for 5–10 seconds) from 50.9% to 60%. Improvements were also evident in broader asthma management practices. For example, Q10 (conducting a thorough asthma history) “often” responses increased from 16.4% to 31%, Q11 (identifying modifiable risk factors) from 24.5% to 60%, Q12 (checking for a written asthma action plan) from 20.9% to 49%, Q13 (examining inhalation technique) from 38.2% to 52%, and Q14–Q15 (assessing patient preferences and side effects) from 36.4–50.9% to 50–59%. Other items also improved: Q17 (evaluating adherence) from 33.6% to 50%, Q18 (advising regular inhaled corticosteroid use) from 50% to 58%, Q19 (counseling before medication discontinuation) from 50% to 56%, Q20 (teaching self-monitoring) from 32.7% to 41%. Q21 (evaluating symptom control over the last 4 weeks) “Sometimes” responses increased from 50% to 78% post-intervention. Finally, Q22 (follow-up scheduling for controlled asthma patients), which was rarely performed pre-intervention, improved after the workshop. “Never” responses decreased from 18.2% to 2%, “rarely” from 43.6% to 23%, “sometimes” increased from 37.3% to 70%, “often” from 0.9% to 4%, and “always” from 0% to 1% (S2 Fig).

Pre- and post-intervention changes in DPI counseling practices across items Q1–Q22. DPI-specific technique items demonstrated clear improvements, with responses shifting toward “often” and “always” after the workshop. Specifically, Q1 (preparing the inhaler) “often” responses increased from 42.7% pre-intervention to 58% post-intervention, Q2 (breathe out completely) from 43.6% to 52%, Q3 (hold inhaler upright) from 48.2% to 53%, Q4 (mouthpiece placement) from 50.9% to 53%, Q5 (inhale forcefully and deeply) from 50.9% to 53%, Q6 (continue forceful inhalation until full dose) from 52.7% to 53%, Q7 (remove inhaler with closed lips) from 49.1% to 53%, Q8 (hold breath 5–10 seconds) from 50% to 52%, and Q9 (prepare next dose) from 51.8% to 53%. General asthma management practices also improved. Q10 (conduct thorough asthma history) “often” responses increased from 16.4% to 31%, Q11 (identify modifiable risk factors) from 24.5% to 60%, Q12 (check written asthma action plan) from 20.9% to 49%, Q13 (examine inhalation technique) from 38.2% to 52%, Q14 (ask about patient preferences) from 36.4% to 50%, Q15 (ask about treatment side effects) from 50.9% to 59%, Q16 (ensure proper technique adherence) from 51.8% to 62%, Q17 (evaluate adherence) from 33.6% to 50%, Q18 (advise regular ICS use) from 50% to 58%, Q19 (counsel before medication discontinuation) from 50% to 56%, Q20 (teach symptom self-monitoring) from 32.7% to 41%, Q21 (evaluate symptom control over 4 weeks) from 50% to 78%. Finally, Q22 (follow-up scheduling for controlled asthma patients) showed a substantial improvement: “never” decreased from 18.2% to 2%, “rarely” from 43.6% to 23%, “sometimes” increased from 37.3% to 70%, “often” from 0.9% to 4%, and “always” from 0% to 1% (S3 Fig).

### 3.7 Pre and Post educational intervention comparison of knowledge, attitude, practice (KAP) scores among participants using per-protocol and intention-to-treat analyses

Baseline comparison between completers and non-completers showed no significant differences in most variables, including age, gender, workload, education, and pharmacy type (all p > 0.05). A significant difference was observed only in the number of patients served per day (p = 0.003) (S13 Table).

Table 2 shows that the median knowledge score of participants increased significantly from pre- to post-intervention. In the per-protocol analysis, the median score improved from 5 (IQR 4–6) to 6 (IQR 5–7) (Wilcoxon signed-rank test, p < 0.001, effect size r(rb) = 0.883). Similarly, in the intention-to-treat analysis, the median increased from 5 (IQR 4–6) to 6 (IQR 5–7) (p < 0.001, r_rb_ = 0.835).

**Table 2:** Pre-Post educational intervention comparison of knowledge, attitude, practice (KAP) scores among participants using per-protocol and intention-to-treat analyses.

Parameter	Analysis		Median (IQR)	Test Statistics	P-Value	Effect size (rank-biserial correlation, r(rb))
**Knowledge Score**	Per-protocol (n = 100)	Pre	5(4-6)	Z = −5.667	<0.001	0.883
		Post	6 (5-7)			
	ITT (multiple imputations, pooled, N = 110)	Pre	5(4-6)	Z = −4.95	<0.001	0.835
		Post	6 (5-7)			
**Attitude score**	Per-protocol (n = 100)	Pre	19 (18-21)	Z = −6.354	<0.001	0.776
		Post	21 (20-22)			
	ITT (multiple imputations, pooled, N = 110)	Pre	19 (18-20)	Z = −7.82	<0.001	0.757
		Post	21 (20-22)			
**Practice score (MDI)**	Per-protocol (n = 100)	Pre	77 (65-82)	Z = −6.498	<0.001	0.832
		Post	80 (69.25-84)			
	ITT (multiple imputations, pooled, N = 110)	Pre	76 (65-82)	Z = −5.08	<0.001	0.823
		Post	80 (70-84)			
**Practice score (DPI)**	Per-protocol (n = 100)	Pre	75 (65-82)	Z = −7.699	<0.001	1.0
		Post	80 (67-84)			
	ITT (multiple imputations, pooled, N = 110)	Pre	74 (65-82)	Z = −6.15	<0.001	0.946
		Post	80 (67-84)			

MDI, Metered dose inhalers; DPI, dry powder inhalers; ITT, intention-to-treat analyses using multiple imputation. Effect sizes (r) represent rank-based measures of pre–post change.

Moreover, the median attitude score of participants increased significantly from pre- to post-intervention. In the per-protocol analysis, the median score improved from 19 (IQR 18–21) to 21 (IQR 20–22) (Wilcoxon signed-rank test, p < 0.001, effect size r(rb) = 0.776). Similarly, in the intention-to-treat analysis, the median increased from 19 (IQR 18–20) to 21 (IQR 20–22) (p < 0.001, r(rb)= 0.757).

In addition, the median MDIs practice score of participants increased significantly from pre- to post-intervention. In the per-protocol analysis, the median score improved from 77 (IQR 65–82) to 80 (IQR 69.25–84) (Wilcoxon signed-rank test, p < 0.001, effect size r(rb) = 0.832). Similarly, in the intention-to-treat analysis, the median increased from 76 (IQR 65–82) to 80 (IQR 70–84) (p < 0.001, r(rb)= 0.823).

Similarly, the median DPIs practice score of participants increased significantly from pre- to post-intervention. In the per-protocol analysis, the median score improved from 75 (IQR 65–82) to 80 (IQR 67–84) (Wilcoxon signed-rank test, p < 0.001, effect size r(rb) = 1.0). Similarly, in the intention-to-treat analysis, the median increased from 74 (IQR 65–82) to 80 (IQR 67–84) (p < 0.001, r(rb)= 0.946).

## 4. Discussion

This quasi-experimental intervention study observed improvements across all assessed pharmacists’ KAP domains after the educational workshop. Their knowledge, attitudes, and counseling practices all improved. The improvements were consistent under both per-protocol and intention-to-treat analytical frameworks. In addition, self-reported frequency of counseling behaviors for both counseling skills for both MDIs and DPIs increased after the workshop and became more consistent. In short, this focused guideline workshop was associated with measurable short-term improvements in pharmacists’ knowledge, attitudes and device-specific counselling behaviors, supporting the potential value of pharmacist-focused education as a strategy for strengthening asthma care in the community setting.

At the baseline, the pharmacists showed an overall positive attitude toward their role in asthma care, but notable gaps were shown in knowledge and practice measures. Our baseline findings are generally consistent with research conducted in Jordan and similar settings which often reveal that pharmacists are willing to be involved in asthma care. However, they display deficiencies in their knowledge of guidelines-concordant pharmacotherapy and counselling regarding inhaler technique [16,24–26].

Baseline practice scores for both MDI and DPI counseling were moderate but likely concealed important item-level variability. Inhaler technique counseling is a high-impact behavior because even small errors can substantially reduce medication delivery and contribute to persistent symptoms, frequent reliever use, and exacerbations. The literature consistently documents high rates of inhaler misuse among patients and highlights that health professionals themselves may omit or mis-teach critical steps if counseling is not standardized and reinforced [27–29]. This underscores the importance of addressing even subtle deficiencies in the counselling practice.

Notably, lower consistency in baseline practice behaviors relates to actions that require follow-up and continuity of care, such as reassessment to monitor symptom control. These behaviors are crucial in asthma management because control status and risk evolve over weeks to months, and because patients with frequent reliever refills often remain uncontrolled until a clinician explicitly identifies the pattern. However, loop-closing actions are the most sensitive to workflow barriers. If pharmacies lack prompts, documentation templates, or protected counseling time, these actions are the first to be omitted. This explains why baseline practice can appear “adequate” in simple counseling steps but remain weak in systematic follow-up behaviors, a pattern that is widely reflected in quality-improvement studies [11,30,31].

In addition, our findings revealed that pharmacists holding a PharmD degree demonstrated higher levels of knowledge, more positive attitudes, and stronger self-reported inhaler counseling practices compared with those holding a BSc in pharmacy. Additionally, pharmacists in Amman generally performed slightly better than those in Irbid. While experience contributed modestly to the performance, long working hours were associated with less consistent counseling, even among motivated pharmacists. Furthermore, urban practice settings appear to facilitate better counselling performance through differential access to continuing education and differences in staff and patient demands regarding counselling [32]. Overall, these findings are in line with previous studies which reported that education and work environment, including location and workload, seem to influence how ready pharmacists are to support patients with asthma [16,33–36]. Collectively, these findings highlight the contribution of both individual and system level factors in shaping the practice.

The negative relationship between weekly working hours and practice scores for both device types shows that workload is a modifiable systems factor [32]. An increase in competence due to knowledge or training alone will not ensure the reliability of counseling behaviors if pharmacy workflows are not redesigned to allow for brief, standardized counselling interactions [33]. Therefore, educational interventions may be most effective when combined with workflow-supportive strategies.

Based on our findings, the educational workshop is associated with improvements across KAP scores. The per-protocol analysis regarding KAP scores reflects improvements in both understanding and self-reported counseling behavior. The consistency of findings under intention-to-treat analysis further supports that the effect is not just a result of selective follow-up. In addition, the observed large effect sizes are comparable to those seen in other community pharmacy educational interventions focusing on asthma counseling and inhaler technique [11,37].

Conceptually, this pattern is consistent with a plausible learning pathway. Education corrected high-impact misconceptions, reinforced pharmacists’ professional confidence and role legitimacy, and provided practical procedural skills with a teach-back emphasis [38,39]. This combination is important because knowledge gains alone do not necessarily translate into consistent practice change; however, when knowledge is reinforced through continuous education and ongoing engagement, behavior change is more likely to occur and to persist [40]. This emphasizes the importance of sustained, practice-oriented educational approaches.

The item-level knowledge analysis showed significant improvement in appropriate use of inhaled corticosteroid (ICS) in children, as well as the risk associated with using SABA-only therapy. These findings align with previous studies showing that targeted education can effectively address key knowledge gaps related to the guideline recommended asthma management and can successfully correct the misconceptions in asthma management [41,42].

The changes in attitude scores indicate that the intervention reaffirmed the pharmacists’ role, responsibility, and confidence in providing asthma support in line with guidelines [16]. The observed shift in post-intervention responses towards agreement/strong agreement on attitude items coincides with other intervention studies that train participants on tangible tools and communication scripts [34]. This suggests that confidence-building is an important component of effective educational intervention.

The modifications to MDI and DPI practice score advising values suggest that this intervention increased pharmacists’ ability to deliver device-specific inhaler counseling practices. Researchers consider this an important clinical achievement as inhaler misuse is one of the most common and correctable causes of poor asthma control [39,43]. The intervention focused on technique steps where errors were recurrent and focused on teach-back, which helps retain the technique and reduces critical errors across device types [29,44,45].

These findings are consistent with previous findings by Basheti et al. (2007), who demonstrated that a simple inhaler technique intervention delivered by community pharmacists improved asthma outcomes, supporting the premise that brief, structured counseling at the point of care can yield measurable benefits [46]. In addition, their subsequent work reported that pharmacists’ inhaler technique demonstration skills can be maintained over time with structured training and follow-up, which supports the need for periodic refreshers rather than one-off education [47]. Moreover, a randomized trial found that reminder labels improved retention of inhaler technique skills, illustrating how low-friction workflow tools can strengthen sustainability and reduce skill decay [48]. Evidence from Jordan further supports the local relevance: inhaler technique education among patients hospitalized for asthma was associated with improved technique and better asthma control after discharge, underscoring the magnitude of correctable technique problems and the potential impact of structured education in this setting [49].

Even though these results confirm the intervention’s efficacy, it is important to analyze the degree of change carefully. Although the observed changes in practice scores were statistically significant, their absolute magnitude may appear modest relative to the total scale range. For example, the median MDI practice score increased by a small number of points on a 110-point scale. However, this should be interpreted in the context of the scale structure, which includes a wide range of behaviors, some of which were already performed at relatively high frequency at baseline, suggesting a potential ceiling effect. In addition, the consistent improvements across multiple domains, along with moderate to large effect sizes, indicate that the observed changes are meaningful at a behavioral level. Importantly, even small improvements in inhaler technique counseling may have clinically relevant implications, as improper inhaler use is a well-established contributor to poor asthma control. It should also be noted that a minimum clinically important difference (MCID) has not been established for this KAP instrument, which limits the ability to define a precise threshold for clinical significance.

Despite these factors, one of the strengths of this study is the use of a validated KAP instrument that evaluated the outcome across different domains, including two devices specific counselling practice scores (MDI and DPI). Furthermore, the presence of both per-protocol and intention-to-treat analyses reinforces the robustness of the observed effect of the intervention, alleviating concerns that the improvements may have been selectively followed-up.

## 5. Limitations

This study has several limitations. We used a quasi-experimental design, which allowed us to compare pharmacists’ performance before and after the workshop, but the use of a single-arm pre–post design without a control group limits the ability to draw causal inferences. Future studies employing controlled designs are needed to better establish causality. Additionally, the absence of a control group means that some improvements may be due to other factors, such as familiarity with the questionnaire or awareness of being observed. Questionnaire test–retest reliability was not assessed due to the short follow-up interval and the cross-sectional design, and content validity was evaluated qualitatively based on expert feedback without calculating a quantitative Content Validity Index (CVI).

The two-week follow-up period was likely sufficient to detect changes in knowledge, but it may have been too short to capture meaningful changes in routine practice. The modest practice score may reflect short term recall of the workshop content rather than lasting behavioral changes. Future studies are needed to incorporate follow-up assessment over a long period. In addition, some counseling behaviors were self-reported, which may have led participants to overestimate their performance. Compared with the pilot results, reliability was slightly lower in the full sample for both knowledge and attitude. This is likely due to greater variation among participants in the larger sample and the fact that these scales measure multiple aspects of knowledge and attitude. In applied health research, Cronbach’s alpha values between 0.60 and 0.70 are generally considered acceptable for adapted or relatively short instruments [50,51].

Furthermore, in our study, the pharmacist was recruited through Facebook and directed into a private WhatsApp group, in addition to that, pharmacists who were willing to attend two-hour workshop were included. This recruitment strategy could likely overrepresent pharmacists who are digitally active and interested in asthma care, which may introduce selection bias. As a result, the baseline KAP findings may not be generalizable to all community pharmacists in Jordan, as practice settings, staffing levels, and access to training vary. Although the workshop followed the standardized format across the sessions, potential contamination between sessions may have occurred, which could have affected the responses. Although the observed effect sizes were moderate to large, they should be interpreted with caution given the quasi-experimental pre–post design without a concurrent control group. Such effect sizes may be influenced by non-specific factors, including testing effects, increased awareness due to participation (Hawthorne effect), and regression to the mean. Therefore, while the magnitude of change suggests meaningful short-term improvements, these findings should not be interpreted as definitive evidence of causal effectiveness.

The final sample may not be fully representative of the broader pharmacist population and future studies are needed to include less motivated and less engaged pharmacists to improve representativeness. Finally, patient outcomes, such as asthma control or exacerbations, were not assessed, so the direct impact of the intervention on patients remains unknown.

## 6. Conclusion

In conclusion, this study shows that a short-term guideline-based educational workshop was associated with improvements in knowledge, attitudes and practices among community pharmacists. Pharmacists became more confident in asthma treatment guidelines, and in self-reported frequency of MDI and DPI counseling behaviors. Education was clearly the strongest predictor of KAP scores. These findings suggest that short-term structured educational interventions are feasible and may have short-term educational value in this setting. However, a randomized controlled trial (RCT) with longer follow-up periods should be used to assess whether improvements in knowledge and counseling skills persist over time. It would also be useful to measure patient outcomes, such as asthma control, inhaler technique, exacerbation, and healthcare visits, to see if better pharmacist education helps patients.

## Supporting information

S1 Fig Pre-Post- intervention responses of participants’ attitude in asthma management. (DOCX)

S2 Fig Pre-post educational intervention responses of participants’ practice in asthma management using metered dose inhaler (MDI) counseling. (DOCX)

S3 Fig Pre-Post educational intervention responses of participants’ practice in asthma management using dry powder inhaler (DPI) counseling. (DOCX)

S1 Table Pre- intervention assessment of participants’ knowledge about asthma management (n = 110). (DOCX)

S2 Table Pre- intervention assessment of participants’ attitude towards asthma management (n = 110). (DOCX)

S3 Table Pre- intervention assessment of participants’ practice in asthma management using metered dose inhaler (MDI) counseling (n = 110). (DOCX)

S4 Table Pre- intervention assessment of participants’ practice in asthma management using dry powder inhaler (DPI) counseling (n = 110). (DOCX)

S5 Table Univariate analysis for factors affecting the pre-intervention knowledge score. (DOCX)

S6 Table Univariate analysis for factors affecting the pre-intervention attitude score. (DOCX)

S7 Table Univariate analysis for factors affecting the pre-intervention practice score for metered dose inhalers (MDI). (DOCX)

S8 Table Univariate analysis for factors affecting the pre-intervention practice score for dry powder inhalers (DPI). (DOCX)

S9 Table Poisson regression for knowledge about asthma management therapy. (DOCX)

S10 Table Linear regression predictors of attitude score. (DOCX)

S11 Table Linear regression predictors of metered dose inhalers (MDI) practice score. (DOCX)

S12 Table Linear regression predictors of dry powder inhalers (DPI) practice score. (DOCX)

S13 Table Sociodemographic and basic data of the Completer and Non-Completer Participants. (DOCX)
