# Supplementary material for: The impact of brief educational intervention on Jordanian community pharmacists’ knowledge, attitudes, and practices in asthma management: A quasi-experimental study
Source: PLoS One. 2026 Aug 27;21(8):e0356821. doi: 10.1371/journal.pone.0356821 (PMC13521412; doi:10.1371/journal.pone.0356821)
Supplement: Supplementary file 1 [file pone.0356821.s001.docx]

**S1 Fig.:** **Pre-Post- intervention responses of participants’ attitude in asthma management.**

Detailed question statements are provided in S2 Table.
