# Supplementary material for: The impact of brief educational intervention on Jordanian community pharmacists’ knowledge, attitudes, and practices in asthma management: A quasi-experimental study
Source: PLoS One. 2026 Aug 27;21(8):e0356821. doi: 10.1371/journal.pone.0356821 (PMC13521412; doi:10.1371/journal.pone.0356821)
Supplement: Supplementary file 2 [file pone.0356821.s002.docx]

**S2 Fig: Pre-post educational intervention responses of participants’ practice in asthma management using metered dose inhaler (MDI) counseling.**

Detailed question statements are provided in S3 Table.
