# Supplementary material for: The impact of brief educational intervention on Jordanian community pharmacists’ knowledge, attitudes, and practices in asthma management: A quasi-experimental study
Source: PLoS One. 2026 Aug 27;21(8):e0356821. doi: 10.1371/journal.pone.0356821 (PMC13521412; doi:10.1371/journal.pone.0356821)
Supplement: Supplementary file 4 [file pone.0356821.s004.docx]

**S1 Table: Pre- intervention assessment of participants’ knowledge about asthma management (n=110).**

| **N** | **Question** | **True Answer %** | **False Answer %** |
| --- | --- | --- | --- |
| 1 | Using Steroid inhalation can significantly affect child’s growth | 40.9 | 59.1 |
| 2 | Asthma treatment guidelines no longer recommends short-acting beta2-agonist (SABA) treatment alone. It does not recommend the use of SABA as first choices, it recommends using long-acting beta2-agonist (formoterol) and inhaled corticosteroids (Budesonide) as first choice reliever of symptoms in asthma adults. | 51.8 | 48.2 |
| 3 | Inhaled corticosteroids (ICS) have different doses (low, medium, and high) | 64.5 | 35.5 |
| 4 | SABA-only therapy raises the danger of life-threatening exacerbations and deaths due to asthma | 40.0 | 60.0 |
| 5 | Factors like Poor technique, non-adherence led to poor asthma control | 84.5 | 15.5 |
| 6 | Asthma can be triggered by different factors (eg, Sinus infections, allergies and GERD) | 90.0 | 10.0 |
| 7 | To reduce the risk of infection transmission during epidemics, patients should avoid using nebulizers as much as possible | 87.3 | 12.7 |
|  | Pre-intervention Total Knowledge Score (Median (IQR)) | 5 (4-6) | |
