# Supplementary material for: The impact of brief educational intervention on Jordanian community pharmacists’ knowledge, attitudes, and practices in asthma management: A quasi-experimental study
Source: PLoS One. 2026 Aug 27;21(8):e0356821. doi: 10.1371/journal.pone.0356821 (PMC13521412; doi:10.1371/journal.pone.0356821)
Supplement: Supplementary file 5 [file pone.0356821.s005.docx]

**S2 Table: Pre- intervention assessment of participants’ attitude towards asthma management (n=110).**

| **N** | **Question** | **Strongly disagree** | **Disagree** | **Neutral** | **Agree** | **Strongly Agree** |
| --- | --- | --- | --- | --- | --- | --- |
|  |  | **%** | **%** | **%** | **%** | **%** |
| 1 | The pharmacist is an essential member of the asthma care team | 0 | 0 | 22.7 | 49.1 | 28.2 |
| 2 | I believe I am able to manage asthma patient appropriately | 0 | 24.5 | 33.6 | 38.2 | 3.6 |
| 3 | To be a competent asthma educator, pharmacists must participate in additional continuing medical education programs | 0.9 | 0.9 | 6.4 | 59.1 | 32.7 |
| 4 | Asthmatic patients may benefit from disease monitoring with peak flow meter | 0 | 0.9 | 37.3 | 46.4 | 15.5 |
| 5 | Appropriate asthmatic patient counseling has a significant impact on asthma management success | 0.9 | 0.9 | 12.7 | 68.2 | 17.3 |
|  | **Pre-intervention Total Attitude Score (Median (IQR))** | **19 (18-21)** | | | | |
