# Supplementary material for: The impact of brief educational intervention on Jordanian community pharmacists’ knowledge, attitudes, and practices in asthma management: A quasi-experimental study
Source: PLoS One. 2026 Aug 27;21(8):e0356821. doi: 10.1371/journal.pone.0356821 (PMC13521412; doi:10.1371/journal.pone.0356821)
Supplement: Supplementary file 6 [file pone.0356821.s006.docx]

**S3 Table: Pre- intervention assessment of participants’ practice in asthma management using metered dose inhaler (MDI) counseling (n=110).**

| **N** | **Question** | **Never** | **Rarely** | **Sometimes** | **Often** | **Always** |
| --- | --- | --- | --- | --- | --- | --- |
|  |  | **%** | **%** | **%** | **%** | **%** |
| 1 | Prepare the Inhaler: Shake the inhaler well and remove the cap. | 0 | 1.8 | 49.1 | 48.2 | 0.9 |
| 2 | Breathe out slowly and completely | 0 | 1.8 | 44.5 | 51.8 | 1.8 |
| 3 | Hold the inhaler in upright position | 0 | 1.8 | 44.5 | 51.8 | 1.8 |
| 4 | Insert the mouthpiece into mouth between closed lips | 0 | 1.8 | 44.5 | 51.8 | 1.8 |
| 5 | Activate Medication: Depress the canister once and inhale slowly and deeply at the same time. | 0 | 0.9 | 45.5 | 51.5 | 1.8 |
| 6 | Complete the Inhalation: Continue to breathe in slowly and deeply to ensure the medication reaches the lungs. | 0 | 1.8 | 44.5 | 51.8 | 1.8 |
| 7 | Remove the inhaler with closed lips | 0 | 0.9 | 45.5 | 51.5 | 1.8 |
| 8 | Hold your breath for 5 to 10 seconds | 0 | 1.8 | 45.5 | 50.9 | 1.8 |
| 9 | Prepare for the Next Dose: Wait for 20 to 30 seconds before starting the second puff. | 0 | 0.9 | 45.5 | 51.8 | 1.8 |
| 10 | I conduct a thorough history examination for asthma | 0 | 6.4 | 77.3 | 16.4 | 0 |
| 11 | I know how to identify modifiable risk factors for poor asthma outcomes | 0 | 0.9 | 74.5 | 24.5 | 0 |
| 12 | I check to see if the asthmatic patient has a written asthma action plan | 0 | 8.2 | 70.9 | 20.9 | 0 |
| 13 | I examine my asthmatic patient’s inhalation technique | 0 | 0.9 | 60.9 | 38.2 | 0 |
| 14 | I ask asthmatic patients about their asthma treatment preferences | 0 | 2.7 | 60.9 | 36.4 | 0 |
| 15 | I ask asthmatic patient about any treatment side effects they may have | 0 | 0.9 | 47.3 | 50.9 | 0.9 |
| 16 | I ensure that asthmatic patients follow all medical instructions, including the use of inhalation techniques. | 0 | 0.9 | 46.4 | 51.8 | 0.9 |
| 17 | I evaluate asthmatic patients regarding their adherence to their prescribed regimens | 0 | 1.8 | 61.8 | 33.6 | 2.7 |
| 18 | I advise asthmatic patients to take their inhaled corticosteroids on a regular basis | 0 | 0.9 | 47.3 | 50 | 1.8 |
| 19 | I advise asthmatic patients to consult me before discontinuing any medication | 0 | 0.9 | 47.3 | 50 | 1.8 |
| 20 | I teach asthmatic patients how to self-monitor their symptoms | 0 | 1.8 | 65.5 | 32.7 | 0 |
| 21 | I evaluate symptoms control over the last 4 weeks | 0.9 | 48.2 | 50 | 0.9 | 0 |
| 22 | I schedule a follow-up visit for asthma patients who have been under control for the last 3 months | 18.2 | 43.6 | 37.3 | 0.9 | 0 |
|  | **Pre-intervention Total Practice Score for MDI (Median (IQR))** | 77 (65-82) | | | | |
