# Supplementary material for: The impact of brief educational intervention on Jordanian community pharmacists’ knowledge, attitudes, and practices in asthma management: A quasi-experimental study
Source: PLoS One. 2026 Aug 27;21(8):e0356821. doi: 10.1371/journal.pone.0356821 (PMC13521412; doi:10.1371/journal.pone.0356821)
Supplement: Supplementary file 7 [file pone.0356821.s007.docx]

**S4 Table: Pre- intervention assessment of participants’ practice in asthma management using dry powder inhaler (DPI) counseling (n=110).**

| **N** | **Question** | **Never** | **Rarely** | **Sometimes** | **Often** | **Always** |
| --- | --- | --- | --- | --- | --- | --- |
|  |  | **%** | **%** | **%** | **%** | **%** |
| **1** | Prepare the Inhaler: Hold the Diskus in one hand, push the thumb grip away from you until it clicks, then slide the lever until it clicks to load the dose | 0 | 1.8 | 54.5 | 42.7 | 0.9 |
| **2** | Breathe out slowly and completely | 0 | 1.8 | 53.6 | 43.6 | 0.9 |
| **3** | Hold the inhaler in upright position | 0 | 1.8 | 50 | 48.2 | 0 |
| **4** | Insert the mouthpiece into mouth between closed lips | 0 | 1.8 | 47.3 | 50.9 | 0 |
| **5** | Inhale forcefully and deeply through the mouth to draw in the powder. | 0 | 0.9 | 48.2 | 50.9 | 0 |
| **6** | Continue the forceful and deep inhalation until the full dose is inhaled. | 0 | 1.8 | 45.5 | 52.7 | 0 |
| **7** | Remove the inhaler with closed lips | 0 | 0.9 | 49.1 | 49.1 | 0.9 |
| **8** | Hold your breath for 5 to 10 seconds | 0 | 1.8 | 48 | 50 |  |
| **9** | Prepare for the Next Dose: Reload the next dose by sliding the lever again. | 0 | 0.9 | 45.5 | 51.8 | 1.9 |
| **10** | I conduct a thorough history examination for asthma | 0 | 6.4 | 77.3 | 16.4 | 0 |
| **11** | I know how to identify modifiable risk factors for poor asthma outcomes | 0 | 0.9 | 74.5 | 24.5 | 0 |
| **12** | I check to see if the asthmatic patient has a written asthma action plan | 0 | 8.2 | 70.9 | 20.9 | 0 |
| **13** | I examine my asthmatic patient’s inhalation technique | 0 | 0.9 | 60.9 | 38.2 | 0 |
| **14** | I ask asthmatic patients about their asthma treatment preferences |  | 2.7 | 60.9 | 36.4 | 0 |
| **15** | I ask asthmatic patient about any treatment side effects they may have | 0 | 0.9 | 47.3 | 50.9 | 0.9 |
| **16** | I ensure that asthmatic patients follow all medical instructions, including the use of inhalation techniques. | 0 | 0.9 | 46.4 | 51.8 | 0.9 |
| **17** | I evaluate asthmatic patients regarding their adherence to their prescribed regimens | 0 | 1.8 | 61.8 | 33.6 | 2.7 |
| **18** | I advise asthmatic patients to take their inhaled corticosteroids on a regular basis | 0 | 0.9 | 47.3 | 50 | 1.8 |
| **19** | I advise asthmatic patients to consult me before discontinuing any medication | 0 | 0.9 | 47.3 | 50 | 1.8 |
| **20** | I teach asthmatic patients how to self-monitor their symptoms | 0 | 1.8 | 65.5 | 32.7 | 0 |
| **21** | I evaluate symptoms control over the last 4 weeks | 0 | 0.9 | 48.2 | 50 | 0.9 |
| **22** | I schedule a follow-up visit for asthma patients who have been under control for the last 3 months | 18.2 | 43.6 | 37.3 | 0.9 | 0 |
|  | **Pre-Intervention Total Practice Score for DPI (Median (IQR))** | 75 (65-82) | | | | |
