# Supplementary material for: The impact of brief educational intervention on Jordanian community pharmacists’ knowledge, attitudes, and practices in asthma management: A quasi-experimental study
Source: PLoS One. 2026 Aug 27;21(8):e0356821. doi: 10.1371/journal.pone.0356821 (PMC13521412; doi:10.1371/journal.pone.0356821)
Supplement: Supplementary file 8 [file pone.0356821.s008.docx]

**S5 Table: Univariate analysis for factors affecting the pre-intervention knowledge score.**

|  | **Median** | **Interquartile range (IQR)** | **Test Statistics** | **P-Value** |
| --- | --- | --- | --- | --- |
| **Gender¥** |  |  | U= 1285.0, Z= -0.894 | 0.371 |
| Males | 5 | 4-6 |  |  |
| Females | 5 | 3.25-6 |  |  |
| **Education background¥** |  |  | U= 502.0, Z= -6.120 | <0.001 |
| Pharmacy | 4 | 2.75-5 |  |  |
| PharmD | 6 | 5-6 |  |  |
| **Post graduate degree¥** |  |  | U= 1054.5, Z= -1.796 | 0.072 |
| Yes | 4 | 3.25-5.75 |  |  |
| No | 5 | 4-6 |  |  |
| **Location of pharmacy¥** |  |  | U= 1145.5, Z= -0.963 | 0.336 |
| Irbid | 4.5 | 3.75-6 |  |  |
| Amman | 5 | 4-6 |  |  |
| Pharmacy type*¥* |  |  | U= 1368.5, Z= -0.863 | 0.388 |
| Chain community pharmacy | 5 | 3-6 |  |  |
| Independent community pharmacy | 5 | 4-6 |  |  |
| Age | r=0.466 | | | <0.001* |
| Working experience (years) | r=0.381 | | | <0.001* |
| Weekly working hours | r= -0.006 | | | 0.947 |
| Number of patients served a day | r=-0.048 | | | 0.381 |
| Number of prescriptions dispensed per day | r=0.212 | | | 0.026* |
| Number of pharmacists working with | r=-0.088 | | | 0.361 |
| Time spent with each patient (in minutes) | r=-0.008 | | | 0.93 |

¥) Mann-Whitney U test; (§) Spearman correlation test was used; *, significant p-value at 0.05 level of significance.
