# Supplementary material for: The impact of brief educational intervention on Jordanian community pharmacists’ knowledge, attitudes, and practices in asthma management: A quasi-experimental study
Source: PLoS One. 2026 Aug 27;21(8):e0356821. doi: 10.1371/journal.pone.0356821 (PMC13521412; doi:10.1371/journal.pone.0356821)
Supplement: Supplementary file 9 [file pone.0356821.s009.docx]

**S6 Table: Univariate analysis for factors affecting the pre-intervention attitude score.**

|  | Median | Interquartile range (IQR) | Test Statistics | P-Value |
| --- | --- | --- | --- | --- |
| Gender¥ |  |  | U= 1269.50, Z= -0.986 | 0.324 |
| Males | 19 | 17-21 |  |  |
| Females | 19 | 18-20 |  |  |
| Education background¥ |  |  | U= 811.0, Z= -4.220 | <0.001 |
| Pharmacy | 18 | 17-20 |  |  |
| PharmD | 20 | 19-21 |  |  |
| Post graduate degree¥ |  |  | U= 1070.0, Z= -1.688 | 0.091 |
| Yes | 18.5 | 17-20 |  |  |
| No | 19 | 18-21 |  |  |
| Location of pharmacy¥ |  |  | U= 1037.0, Z= -1.668 | 0.095 |
| Irbid | 19 | 17-20 |  |  |
| Amman | 19 | 18-21 |  |  |
| Pharmacy type¥ |  |  | U= 1082.50, Z= -2.589 | 0.010 |
| Chain community pharmacy | 20 | 18.5-21 |  |  |
| Independent community pharmacy | 19 | 17-20 |  |  |
| Age | r=0.212 | | | 0.026* |
| Working experience (years) | r=0.255 | | | 0.007* |
| Weekly working hours | r= -0.001 | | | 0.992 |
| Number of patients served a day | r=-0.011 | | | 0.907 |
| Number of prescriptions dispensed per day | r=0.026 | | | 0.791 |
| Number of pharmacists working with | r=-0.094 | | | 0.329 |
| Time spent with each patient (in minutes) | r=-0.17 | | | 0.079 |

¥) Mann-Whitney U test; (§) Spearman correlation test was used; *, significant p-value at 0.05 level of significance.
