# Supplementary material for: The impact of brief educational intervention on Jordanian community pharmacists’ knowledge, attitudes, and practices in asthma management: A quasi-experimental study
Source: PLoS One. 2026 Aug 27;21(8):e0356821. doi: 10.1371/journal.pone.0356821 (PMC13521412; doi:10.1371/journal.pone.0356821)
Supplement: Supplementary file 10 [file pone.0356821.s010.docx]

**S7 Table: Univariate analysis for factors affecting the pre-intervention practice score for metered dose inhalers (MDI).**

|  | **Median** | **Interquartile range (IQR)** | **Test Statistics** | **P-Value** |
| --- | --- | --- | --- | --- |
| Gender¥ |  |  | U= 1187.0, Z= -1.489 | 0.136 |
| Males | 66 | 64-81 |  |  |
| Females | 78 | 65-82 |  |  |
| Education background¥ |  |  | U= 638.0, Z= -5.232 | <0.001 |
| Pharmacy | 65 | 64-78.25 |  |  |
| PharmD | 81 | 68.75-83 |  |  |
| Post graduate degree¥ |  |  | U= 1065.50, Z= -1.705 | 0.088 |
| Yes | 66 | 64.25-79 |  |  |
| No | 77.5 | 65-83 |  |  |
| Location of pharmacy¥ |  |  | U= 983.0, Z= -2.007 | 0.045 |
| Irbid | 65 | 65-81.25 |  |  |
| Amman | 78 | 65-82.75 |  |  |
| Pharmacy type¥ |  |  | U= 1389.0, Z= -0.730 | 0.465 |
| Chain community pharmacy | 78 | 65-83 |  |  |
| Independent community pharmacy | 66 | 65-82 |  |  |
| Age | r=0.322 | | | <0.001* |
| Working experience (years) | r=0.285 | | | 0.003* |
| Weekly working hours | r= -0.199 | | | 0.037* |
| Average number of patients served a day | r=-0.02 | | | 0.837 |
| Average number of prescriptions dispensed per day | r=0.083 | | | 0.389 |
| Number of pharmacists working with | r=-0.029 | | | 0.765 |
| Average time spent with each patient (in minutes) | r=0.043 | | | 0.654 |

¥) Mann-Whitney U test; (§) Spearman correlation test was used; *, significant p-value at 0.05 level of significance.
