# Supplementary material for: The impact of brief educational intervention on Jordanian community pharmacists’ knowledge, attitudes, and practices in asthma management: A quasi-experimental study
Source: PLoS One. 2026 Aug 27;21(8):e0356821. doi: 10.1371/journal.pone.0356821 (PMC13521412; doi:10.1371/journal.pone.0356821)
Supplement: Supplementary file 11 [file pone.0356821.s011.docx]

**S8 Table: Univariate analysis for factors affecting the pre-intervention practice score for dry powder inhalers (DPI).**

|  | Median | Interquartile range (IQR) | Test Statistics | P-Value |
| --- | --- | --- | --- | --- |
| Gender¥ |  |  | U= 1204.50, Z= -1.381 | 0.167 |
| Males | 66 | 64-81 |  |  |
| Females | 76.5 | 65-82 |  |  |
| Education background¥ |  |  | U= 664.50, Z= -5.071 | <0.001 |
| Pharmacy | 65 | 64-78 |  |  |
| PharmD | 79.5 | 68-83 |  |  |
| Post graduate degree¥ |  |  | U= 1054.0, Z= -1.778 | 0.075 |
| Yes | 66 | 64.25-79 |  |  |
| No | 75.5 | 65-82.25 |  |  |
| Location of pharmacy¥ |  |  | U= 956.0, Z= -2.182 | 0.029 |
| Irbid | 65 | 65-79.5 |  |  |
| Amman | 77.5 | 65-82 |  |  |
| Pharmacy type¥ |  |  | U= 1441.50, Z= -0.414 | 0.679 |
| Chain community pharmacy | 76 | 65-82 |  |  |
| Independent community pharmacy | 66 | 65-82 |  |  |
| Age | r=0.342 | | | <0.001* |
| Working experience (years) | r=0.301 | | | 0.001* |
| Weekly working hours | r= -0.206 | | | 0.031 |
| Average number of patients served a day | r=-0.004 | | | 0.967 |
| Average number of prescriptions dispensed per day | r=0.085 | | | 0.377 |
| Number of pharmacists working with | r=-0.06 | | | 0.531 |
| Average time spent with each patient (in minutes) | r=0.025 | | | 0.789 |

¥) Mann-Whitney U test; (§) Spearman correlation test was used; *, significant p-value at 0.05 level of significance.
