# Supplementary material for: The impact of brief educational intervention on Jordanian community pharmacists’ knowledge, attitudes, and practices in asthma management: A quasi-experimental study
Source: PLoS One. 2026 Aug 27;21(8):e0356821. doi: 10.1371/journal.pone.0356821 (PMC13521412; doi:10.1371/journal.pone.0356821)
Supplement: Supplementary file 12 [file pone.0356821.s012.docx]

**S9 Table: Poisson regression for knowledge about asthma management therapy.**

|  | **Beta** | **SE** | **95%**  **Wald CI** | | **Wald χ^2^** | **RR** | **p-value** |
| --- | --- | --- | --- | --- | --- | --- | --- |
| **(Intercept)** | 1.466 | 0.5142 | 0.458 | 2.474 | 8.129 | 4.33 | 0.004 |
| **Gender (Female vs Male)** | -0.056 | 0.0964 | -0.245 | 0.133 | 0.338 | 0.95 | 0.561 |
| **Education (Pharmacy vs PharmD)** | -0.414 | 0.1035 | -0.617 | -0.211 | 16.021 | 0.66 | <0.001 |
| **Postgraduate (Yes vs No)** | -0.041 | 0.1075 | -0.252 | 0.170 | 0.143 | 0.96 | 0.705 |
| **Location (Amman vs Irbid)** | -0.065 | 0.1031 | -0.267 | 0.137 | 0.402 | 0.94 | 0.526 |
| **Pharmacy type**  **(Chain Community Pharmacy vs**  **independent community pharmacy)** | 0.011 | 0.0960 | -0.177 | 0.199 | 0.013 | 1.01 | 0.909 |
| **Working experience (years)** | 0.015 | 0.0122 | -0.009 | 0.039 | 1.457 | 1.02 | 0.227 |
| **Weekly working hours** | 0.004 | 0.0077 | -0.011 | 0.019 | 0.319 | 1.00 | 0.572 |
| **Average number of prescriptions**  **dispensed per day** | 0.012 | 0.0105 | -0.008 | 0.033 | 1.352 | 1.01 | 0.245 |
| **Average time spent with each patient**  **(in minutes)** | -0.020 | 0.0222 | -0.063 | 0.024 | 0.780 | 0.98 | 0.377 |

CI, confidence interval; RR, rate ratio; SE, standard error; *, significant p-value at 0.05 using Wald test.
