# Supplementary material for: The impact of brief educational intervention on Jordanian community pharmacists’ knowledge, attitudes, and practices in asthma management: A quasi-experimental study
Source: PLoS One. 2026 Aug 27;21(8):e0356821. doi: 10.1371/journal.pone.0356821 (PMC13521412; doi:10.1371/journal.pone.0356821)
Supplement: Supplementary file 13 [file pone.0356821.s013.docx]

**S10 Table: Linear regression predictors of attitude score.**

| **Parameter** | **Beta** | **SE** | **t-statistic** | **p-value** |
| --- | --- | --- | --- | --- |
| **(Constant)** | 14.376 | 2.736 | 5.254 | <0.001 |
| **Gender (Female vs Male)** | -0.568 | 0.552 | -1.030 | 0.305 |
| **Education (Pharmacy vs PharmD)** | 1.326 | 0.580 | 2.286 | 0.024 |
| **Postgraduate (Yes vs No)** | -0.725 | 0.588 | -1.233 | 0.220 |
| **Location (Amman vs Irbid)** | 0.470 | 0.577 | 0.815 | 0.417 |
| **Pharmacy type**  **(Chain Community Pharmacy vs**  **independent community pharmacy)** | -0.700 | 0.537 | -1.305 | 0.195 |
| **Working experience (years)** | 0.111 | 0.072 | 1.547 | 0.125 |
| **Weekly working hours** | 0.068 | 0.042 | 1.596 | 0.114 |
| **Average number of prescriptions**  **dispensed per day** | 0.017 | 0.057 | 0.302 | 0.764 |
| **Average time spent with each patient**  **(in minutes)** | 0.047 | 0.122 | 0.386 | 0.700 |

Beta, regression coefficient; SE, standard error; *, significant p-value at 0.05 level of significance.
