# Supplementary material for: The impact of brief educational intervention on Jordanian community pharmacists’ knowledge, attitudes, and practices in asthma management: A quasi-experimental study
Source: PLoS One. 2026 Aug 27;21(8):e0356821. doi: 10.1371/journal.pone.0356821 (PMC13521412; doi:10.1371/journal.pone.0356821)
Supplement: Supplementary file 14 [file pone.0356821.s014.docx]

**S11 Table: Linear regression predictors of metered dose inhalers (MDI) practice score.**

| **Parameter** | **Beta** | **SE** | **t-statistic** | **p-value** |
| --- | --- | --- | --- | --- |
| **(Constant)** | 76.585 | 7.912 | 9.680 | <0.001 |
| **Gender (Female vs Male)** | -2.305 | 1.596 | -1.445 | 0.152 |
| **Education (Pharmacy vs PharmD)** | 6.670 | 1.677 | 3.979 | <0.001 |
| **Postgraduate (Yes vs No)** | -1.471 | 1.701 | -0.865 | 0.389 |
| **Location (Amman vs Irbid)** | 4.618 | 1.667 | 2.770 | 0.007 |
| **Pharmacy type**  **(Chain Community Pharmacy vs**  **independent community pharmacy)** | -0.709 | 1.552 | -0.457 | 0.649 |
| **Working experience (years)** | 0.421 | 0.207 | 2.034 | 0.045 |
| **Weekly working hours** | -0.174 | 0.122 | -1.419 | 0.159 |
| **Average number of prescriptions**  **dispensed per day** | -0.075 | 0.165 | -0.451 | 0.653 |
| **Average time spent with each patient**  **(in minutes)** | -0.050 | 0.352 | -0.141 | 0.888 |

Beta, regression coefficient; SE, standard error; *, significant p-value at 0.05 level of significance.
