# Supplementary material for: The impact of brief educational intervention on Jordanian community pharmacists’ knowledge, attitudes, and practices in asthma management: A quasi-experimental study
Source: PLoS One. 2026 Aug 27;21(8):e0356821. doi: 10.1371/journal.pone.0356821 (PMC13521412; doi:10.1371/journal.pone.0356821)
Supplement: Supplementary file 15 [file pone.0356821.s015.docx]

**S12 Table: Linear regression predictors of dry powder inhalers (DPI) practice score.**

| **Parameter** | **Beta** | **SE** | **t-statistic** | **p-value** |
| --- | --- | --- | --- | --- |
| **(Constant)** | 77.485 | 7.634 | 10.150 | <0.001 |
| **Gender (Female vs Male)** | -1.882 | 1.539 | -1.222 | 0.225 |
| **Education (Pharmacy vs PharmD)** | 6.270 | 1.618 | 3.876 | <0.001 |
| **Postgraduate (Yes vs No)** | -1.471 | 1.641 | -0.896 | 0.372 |
| **Location (Amman vs Irbid)** | 4.826 | 1.609 | 3.000 | 0.003 |
| **Pharmacy type**  **(Chain Community Pharmacy vs**  **independent community pharmacy)** | -0.531 | 1.497 | -0.355 | 0.723 |
| **Working experience (years)** | 0.387 | 0.199 | 1.942 | 0.055 |
| **Weekly working hours** | -0.186 | 0.118 | -1.572 | 0.119 |
| **Average number of prescriptions**  **dispensed per day** | -0.072 | 0.160 | -0.453 | 0.651 |
| **Average time spent with each patient**  **(in minutes)** | -0.170 | 0.340 | -0.500 | 0.618 |

Beta, regression coefficient; SE, standard error; *, significant p-value at 0.05 level of significance.
