# Supplementary material for: The impact of brief educational intervention on Jordanian community pharmacists’ knowledge, attitudes, and practices in asthma management: A quasi-experimental study
Source: PLoS One. 2026 Aug 27;21(8):e0356821. doi: 10.1371/journal.pone.0356821 (PMC13521412; doi:10.1371/journal.pone.0356821)
Supplement: Supplementary file 16 [file pone.0356821.s016.docx]

**S13 Table: Sociodemographic and basic data of the Completer and Non-Completer Participants.**

| **Variable** | **Completer (n=100)** | **Non completer (n=10)** | **P value** |
| --- | --- | --- | --- |
| **Age (median (IQR))** | 27 (25-31) | 27 (26-28) | 0.51 |
| **Working experience (years)** | 4 (3-9) | 4 (3.5-5) | 0.533 |
| **Weekly working hours** | 54 (45-54) | 54 (54-54) | 0.292 |
| **Number of patients served a day** | 30 (25-40) | 20 (20-30) | 0.003 |
| **Number of prescriptions dispensed per day** | 10 (10-15) | 10 (5-11.25) | 0.093 |
| **Number of pharmacists working with** | 1 (1-2) | 1 (1-1) | 0.316 |
| **Time spent with each patient (in minutes)** | 5 (5-8.75) | 5 (5-6.25) | 0.287 |
| **Gender** |  |  | 0.177 |
| Males | 36 | 6 |  |
| Females | 64 | 4 |  |
| **Education background** |  |  | 0.328 |
| Pharmacy | 51 | 7 |  |
| Pharm D | 49 | 3 |  |
| **Post graduate degree** |  |  | 0.291 |
| Yes | 31 | 5 |  |
| No | 69 | 5 |  |
| **Location of pharmacy** |  |  | 0.495 |
| Irbid | 30 | 4 |  |
| Amman | 70 | 6 |  |
| **Pharmacy type** |  |  | 1.0 |
| Chain community pharmacy | 48 | 5 |  |
| Independent community pharmacy | 52 | 5 |  |
